# Wenn der Darm die Kilos schluckt

**DOI:** 10.1007/s00108-026-02170-w

**Published:** 2026-07-30

**Authors:** Pavol Mikula, Matthias Unseld, Hans Jürgen Heppner

**Affiliations:** 1 Klinik für Geriatrie und Geriatrische Tagesklinik, Klinikum Bayreuth, Medizinischer Campus Oberfranken (MCO), Preuschwitzer Straße 101, 95448 Bayreuth, Deutschland; 2 Lehrstuhl für Geriatrie, Friedrich-Alexander-Universität Erlangen-Nürnberg, Hugenottenplatz 6, 91054 Erlangen, Deutschland; 3 Abteilung für Klinische Forschung SBG, Akademie für Alternsforschung, Haus der Barmherzigkeit, 1160 Wien, Österreich; 4 Klinische Abteilung für Palliativmedizin, Universitätsklinik für Innere Medizin I, Medizinische Universität Wien, 1090 Wien, Österreich

**Keywords:** Geriatrie, Gewichtsabnahme, Mesenteriale Ischämie, Arteriosklerose, Transluminale Angioplastie, Geriatrics, Weight loss, Mesenteric ischemia, Arteriosclerosis, Transluminal angioplasty

## Abstract

Wir berichten über einen 88-jährigen Patienten mit ungewollter Gewichtsabnahme. Diese verlief subjektiv asymptomatisch und war im Nachgang reversibel. Es wurde eine chronische mesenteriale Ischämie auf dem Boden einer Arteriosklerose als Ursache identifiziert und interventionell behandelt. Im Verlauf zeigte sich eine deutliche Gewichtszunahme, ein eher seltenes Phänomen bei hochbetagten geriatrischen Patienten. Sowohl eine breite und zielgerichtete Ursachenabklärung als auch interventionelle Therapien sind im hohen Lebensalter sicher und durchführbar.

## Abstract (en)

The case of an 88-year-old male patient with progressive, unintentional weight loss is reported. The condition was subjectively asymptomatic and subsequently completely reversible. Following a comprehensive internal medicine examination, chronic mesenteric ischemia due to arteriosclerosis was identified as the cause and treated interventionally. Over time, the patient experienced significant weight gain, a comparatively rare phenomenon in very old geriatric patients. Broad and targeted investigation of the cause and interventional therapies are safe and feasible even in old age.

## Anamnese

Im Mai 2021 wurde unser 88-jähriger Patient wegen einer ungewollten und fortschreitenden Gewichtsabnahme von nahezu 40 kg über drei Jahre, davon 5 kg in den letzten zwei Monaten, stationär aufgenommen. Er klagte über häufige Durchfälle in den letzten Tagen und Inappetenz. Eine diesbezügliche gastroenterologische Abklärung war in den Vormonaten bereits mehrfach ambulant und auch stationär erfolgt. Abgesehen von einer exokrinen Pankreasinsuffizienz, die über einen längeren Zeitraum ohne wesentlichen Erfolg mit Enzymsubstitution behandelt wurde, lieferten diese Untersuchungen keine richtungsweisende Erklärung.

## Befund

**Abb. 1:**
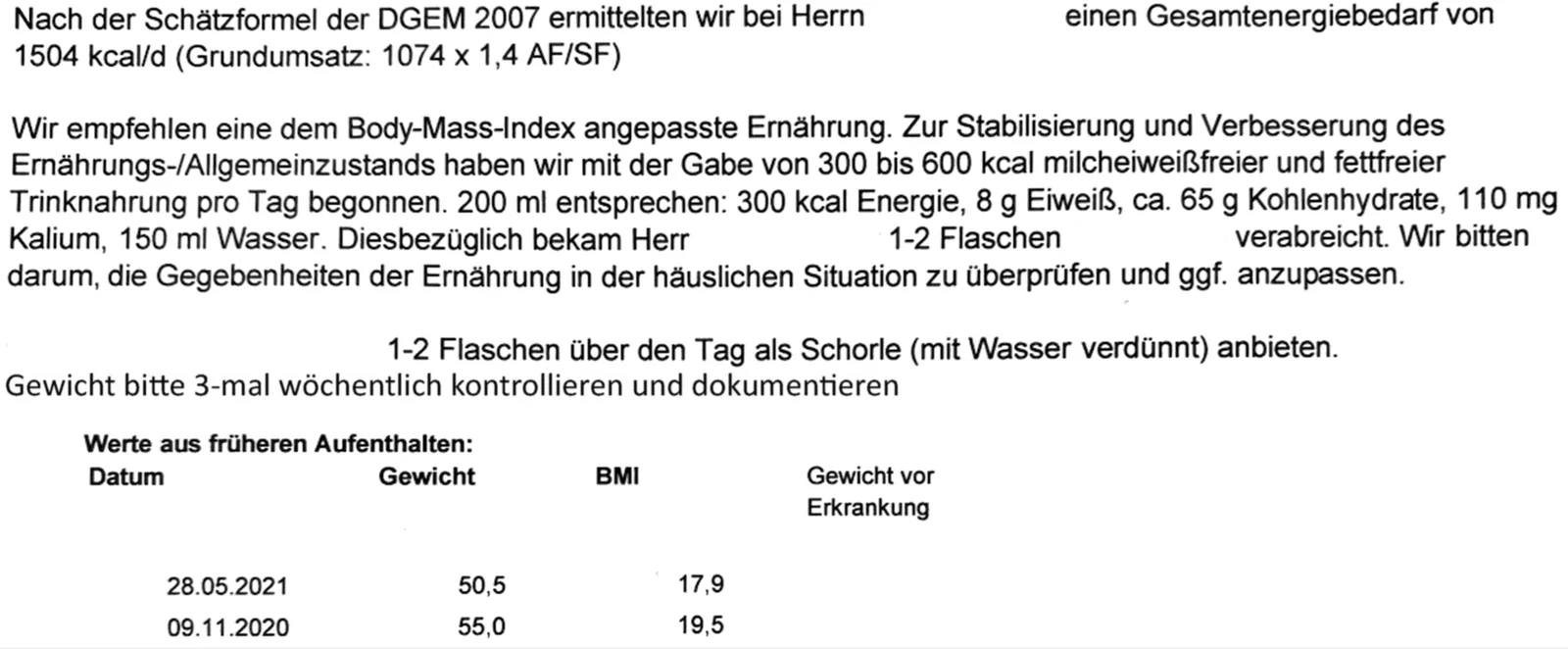
Ernährungsassessment mit Gewicht von 50,5 kg und BMI 17,9 vom Jahr 2021, Auszug aus der Patientenakte

Der Patient befand sich zum Zeitpunkt der Vorstellung in einem guten Allgemeinzustand, jedoch in einem kachektischen Ernährungszustand. Abgesehen von einem leichten diastolischen Geräusch und einer normofrequenten Arrhythmie bei bekanntem Vorhofflimmern war die körperliche Untersuchung unauffällig; es zeigten sich keine Zeichen einer kardiopulmonalen Überwässerung. Das Gewicht bei Vorstellung betrug 50,5 kg, der BMI 17,9 (Abb. 1).

## Diagnose

Zur weiteren Abklärung erfolgte zunächst eine Stuhluntersuchung auf pathogene Keime mittels FilmArray Gastrointestinal (GI) Panel (BioFire Diagnostics, Teil von bioMérieux, Salt Lake City, UT, USA; Tab. 1). Die Dosis der Pankreasenzyme zu den Mahlzeiten wurde ohne wesentlichen Effekt auf die Stuhlfrequenz erhöht.

**Tab. 1:** Ein GI-Panel (Gastrointestinal Panel) ist ein molekulardiagnostischer Stuhltest, der mittels PCR (Polymerase-Kettenreaktion) eine Vielzahl von Erregern gleichzeitig nachweist, die akute Gastroenteritis (Durchfall) verursachen können.

Bakterien	Viren	Parasiten
*Campylobacter* spp. (inkl. *C. jejuni, C. coli, C. upsaliensis*)	Adenovirus F 40/41	*Cryptosporidium* spp.
*Clostridioides difficile* (Toxin A/B)	Astrovirus	*Cyclospora cayetanensis*
*Plesiomonas shigelloides*	Norovirus Genogruppe I/II	*Dientamoeba fragilis*
*Salmonella* spp.	Rotavirus A	*Entamoeba histolytica*
*Shigella* spp./enteroinvasive *Escherichia coli* (EIEC)	Sapovirus (Genogruppen I, II, IV, V)	*Giardia lamblia* (auch als *G. intestinalis* oder *G. duodenalis* bekannt)
*Vibrio* spp. (inkl. *V. parahaemolyticus*, *V. vulnificus*, *V. cholerae*)		*Blastocystis hominis*
*Vibrio cholerae* (spezifisch)		
*Yersinia enterocolitica*		
Enteroaggregative *Escherichia coli* (EAEC)		
Enteropathogene *Escherichia coli* (EPEC)		
Enterotoxische *Escherichia coli* (ETEC)		
Shiga-Toxin-produzierende *Escherichia coli* (STEC) (inkl. *E. coli* O157)		
*Escherichia coli* O157 (spezifisch)		
*Aeromonas* spp.		

**Abb. 2:**
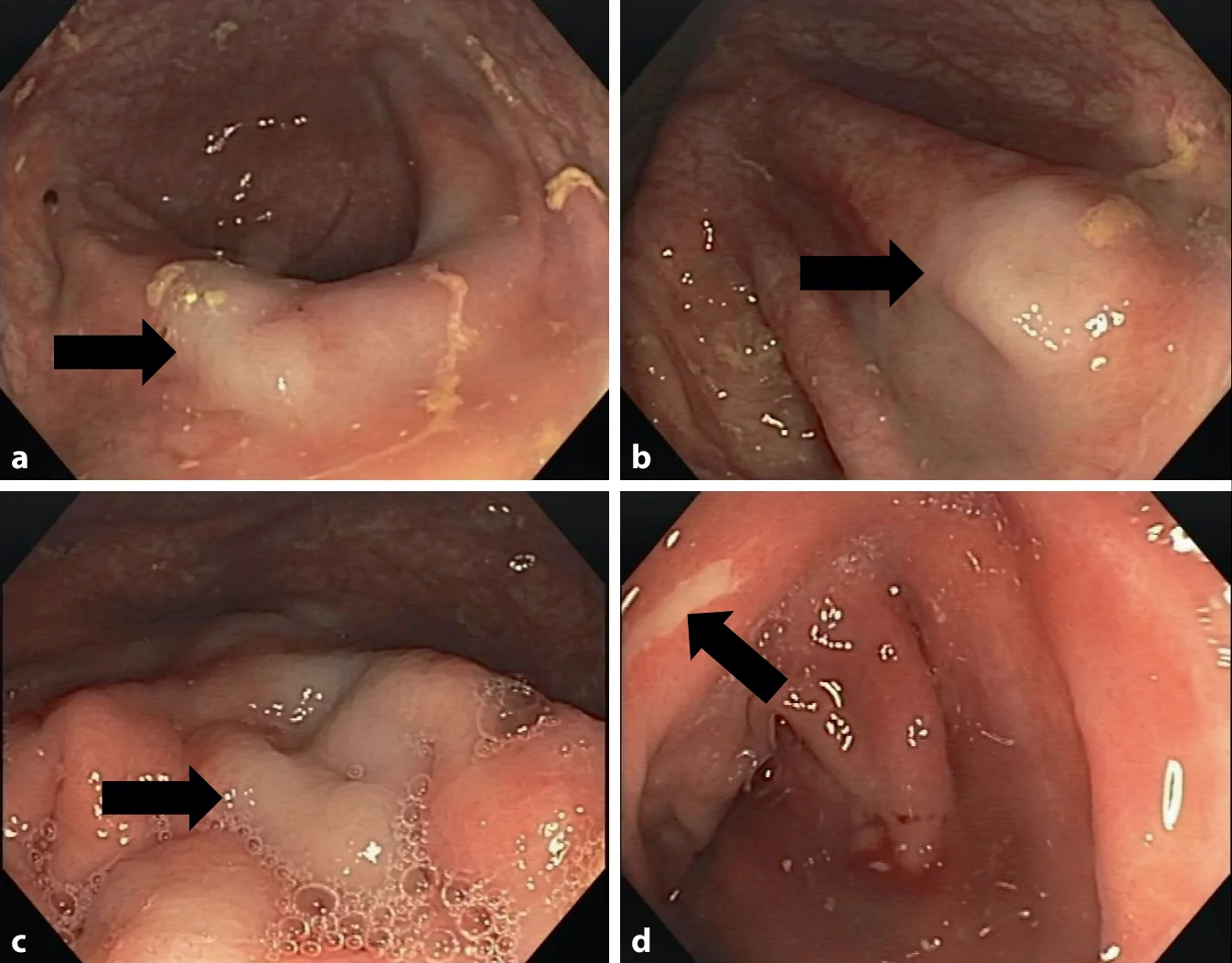
Endoskopische Bilder der Koloskopie mit blassen Arealen sowie zum Teil ischämisch-erosiven Veränderungen (*schwarze Pfeile*)

Aufgrund einer Anämie mit Vitamin-B12-Mangel, leichtem Folsäure- und Eisenmangel sowie einem positiven Test auf okkultes Blut im Stuhl wurden eine endoskopische Diagnostik mittels Ösophagogastroduodenoskopie (ÖGD) und eine Koloskopie durchgeführt. Die ÖGD zeigte sich makroskopisch unauffällig. Bei der Koloskopie fielen im gesamten Kolon sowie im terminalen Ileum stellenweise blasse, makroskopisch hypoperfundierte Areale auf. Diese waren im rechten Colon transversum sowohl quantitativ als auch makroskopisch stärker ausgeprägt als im linken Kolon (Abb. 2). Zusätzlich fanden sich erosive Veränderungen im Bereich des terminalen Ileums sowie ein solitäres, a.e. ischämisches Ulkus im Colon ascendens.

**Abb. 3:**
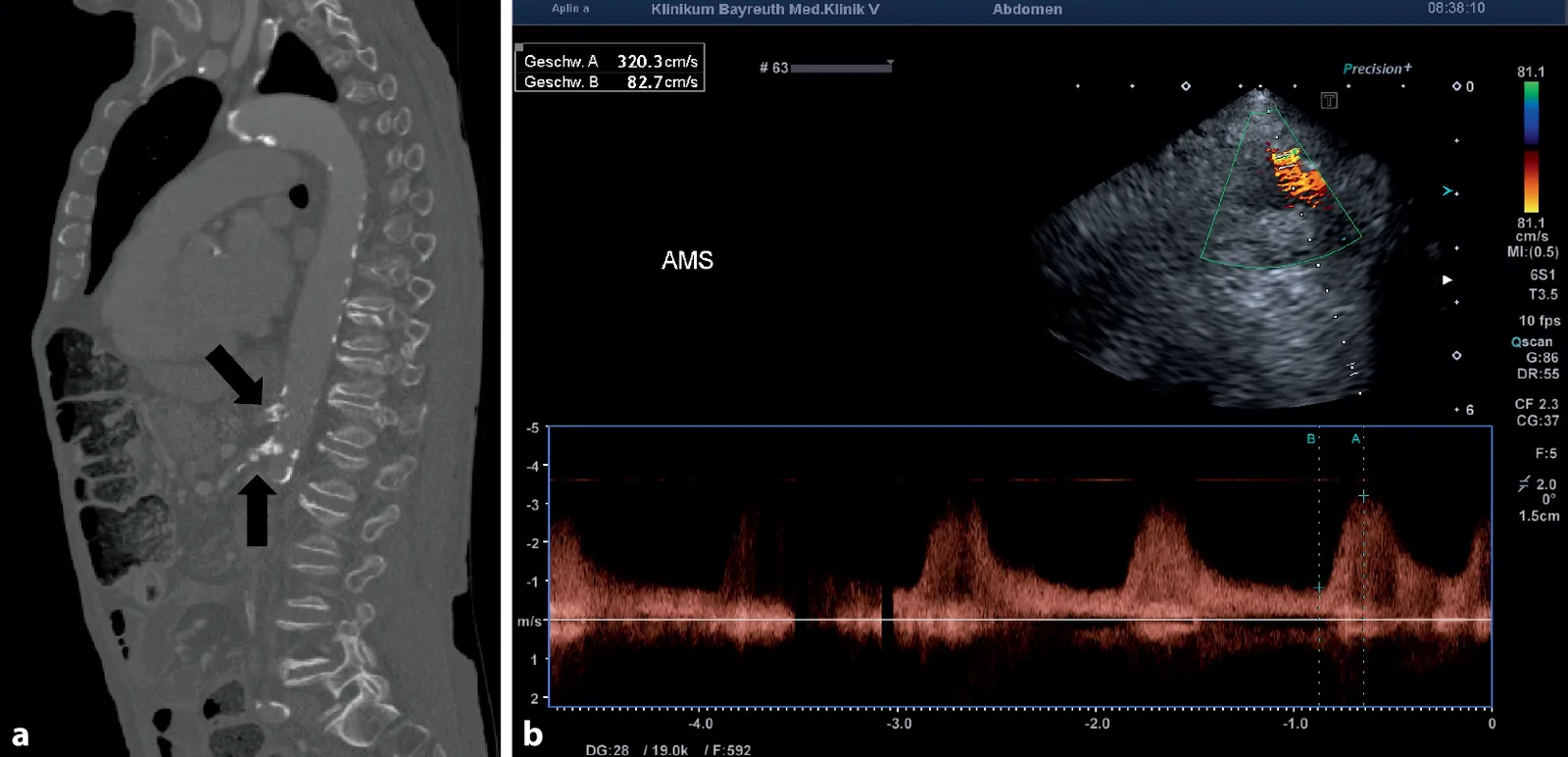
Computertomographie mit Nachweis von Verkalkungen im Bereich der Arteria mesenterica superior sowie im Bereich des Truncus coeliacus im sagittalen Schnitt (**a**). Quantifizierung der Stenose der Arteria mesenterica superior mittels Duplexsonographie (**b**)

Die Histologie der Stufenbiopsien aus dem Duodenum war hinsichtlich eines Morbus Whipple oder einer Zöliakie unauffällig. In den Biopsien aus dem Deszendens, Sigma und Rektum wurde das Bild einer abgelaufenen infektiösen Kolitis beschrieben, sodass bezüglich der akut berichteten Diarrhöen von einer infektiösen Ursache auszugehen war. Es folgte eine Computertomographie des Thorax und Abdomens. Diese zeigte, passend zu unserer klinischen Vermutung, als Nebenbefund eine mittelgradige Abgangsstenose des Truncus coeliacus und eine kurzstreckige hochgradige Stenose der Arteria mesenterica superior (AMS) proximal, welche auch duplexsonographisch bestätigt wurden (Abb. 3).

## Therapie und Verlauf

Chronische mesenteriale Ischämie bei hochgradiger Stenose der Arteria mesenterica superior.

**Abb. 4:**
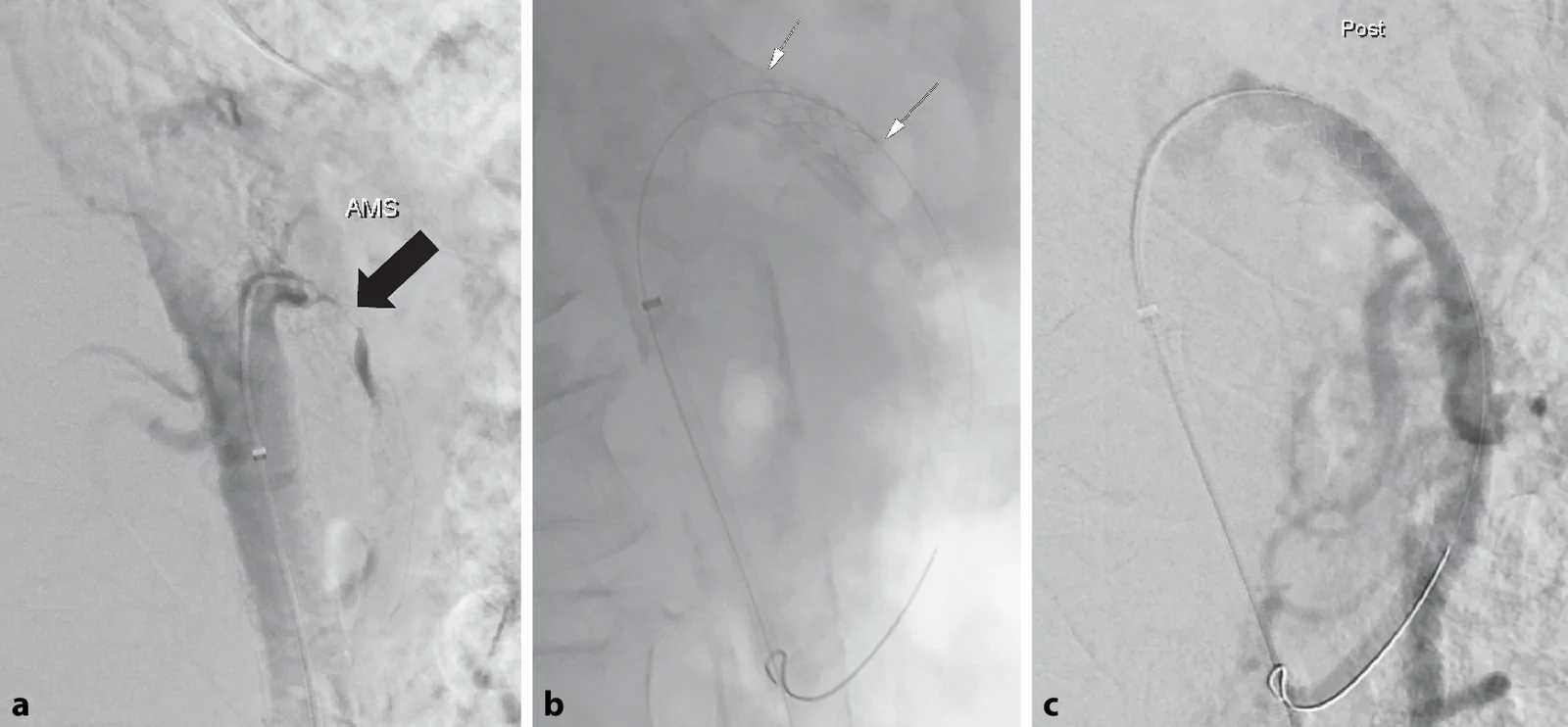
Die diagnostische Angiographie zeigte die Stenose der AMS (*schwarzer Pfeil*, **a**). Im Rahmen der Intervention wurde ein Stent platziert (*weiße Pfeile*, **b**), und postinterventionell war ein guter Fluss sichtbar (**c**). (Mit freundlicher Genehmigung Radiologie Klinikum Bayreuth)

Angesichts der Ergebnisse stellten wir die Indikation zur interventionellen Angiographie. Während des Eingriffs wurde die Arteria femoralis communis rechts punktiert und die Gefäßdarstellung retrograd durchgeführt. Anschließend erfolgte eine erfolgreiche Rekanalisation der Arteria mesenterica superior mittels perkutaner transluminaler Angioplastie (PTA) und Implantation eines 6 mm/2 cm-Stents (PALMAZ BLUE™). Auf Wunsch des Patienten und aufgrund seiner Sorge vor den Risiken einer oralen Antikoagulation wurde trotz des vorliegenden Vorhofflimmerns ein individualisiertes Vorgehen gewählt und eine duale Thrombozytenaggregationshemmung mittels ASS und Clopidogrel für die Dauer von 28 Tagen festgelegt (Abb. 4).

Unser Patient wurde nach der komplikationslosen Intervention Ende Juni 2021 mit einem Gewicht von 51 kg entlassen.

Zwei Jahre später, Ende August 2023, wurde der Patient wegen eines Gichtanfalls am rechten Fuß mit begleitender hypertensiver Krise stationär in der Geriatrie aufgenommen.

**Abb. 5:**
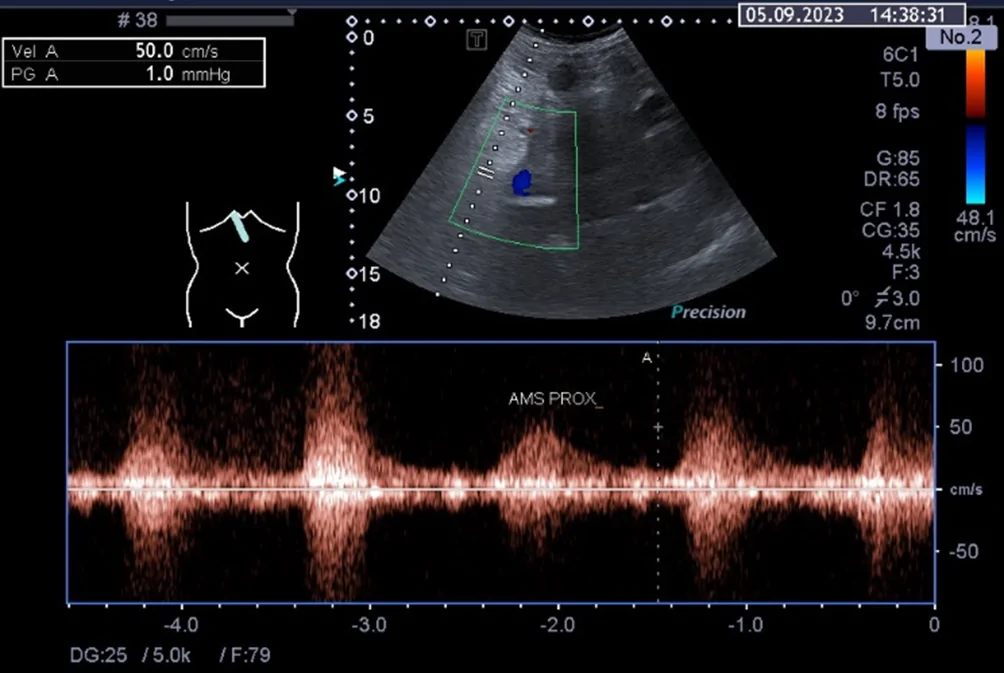
Duplexsonographie der Arteria mesenterica superior mit Flussprofil

**Abb. 6:**
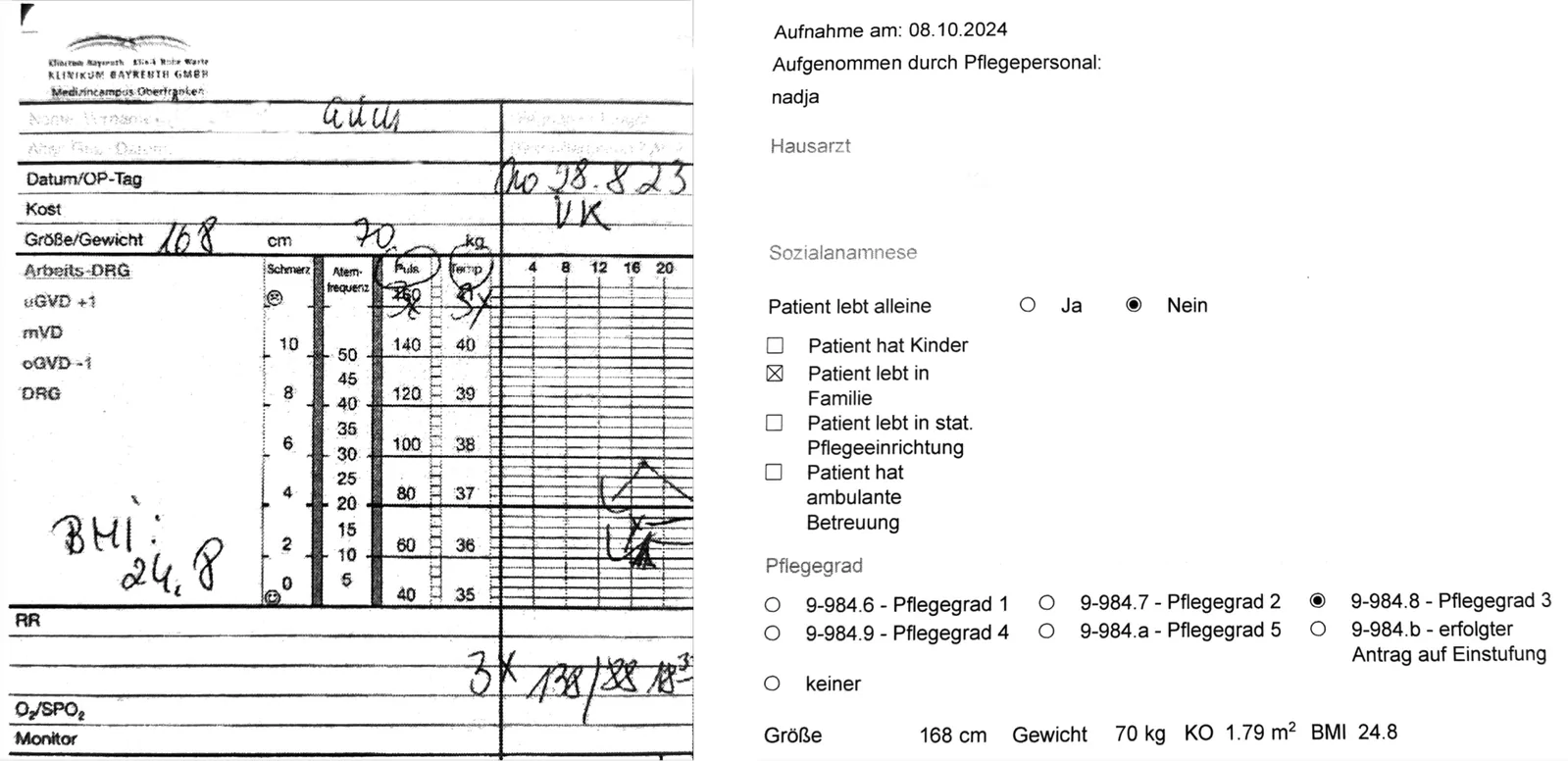
*Links*: Auszug aus der Patientenakte im Jahr 2023 mit damaligem BMI (Auszug aus der Patientenakte). *Rechts*: dokumentiertes Gewicht von 70 kg drei Jahre nach der Intervention (Auszug aus der Patientenakte)

Erfreulicherweise konnten wir bei diesem Aufenthalt ein Gewicht des Patienten von 70 kg dokumentieren. Sonographisch zeigte sich ein unauffälliges Flussprofil der Arteria mesenterica superior (Abb. 5), die Kachexiezeichen hatten sich zurückgebildet, und der Body-Mass-Index (BMI) von 24,8 zeugte von einer Gewichtszunahme von 20 kg (Abb. 6 links).

Auch im dritten Jahr nach der Intervention, im Oktober 2024, wurde unser mittlerweile 91-jähriger Patient aufgrund eines akuten prärenalen Nierenversagens aufgenommen. Das Gewicht betrug weiterhin stabile 70 kg, was von einem nachhaltig stabilen Langzeitergebnis zeugt (Abb. 6 rechts).

## Diskussion

Man unterscheidet zwischen einer akuten und einer chronischen mesenterialen Ischämie. Die akute Form stellt eine seltene, aber potenziell tödliche Gruppe von Darmerkrankungen dar, die unbehandelt eine hohe Sterblichkeitsrate aufweist [1, 2]. Die chronische Form hingegen kann manchmal ohne jegliche Symptomatik vorhanden sein und hat pathophysiologisch eine komplexe Malabsorption zur Folge, wie in dem oben erwähnten Fall.

Die erniedrigten Konzentrationen von Elastase 1 im Stuhl, die sich ambulant feststellen ließen, waren retrospektiv betrachtet aufgrund des fehlenden Therapieerfolgs unter Enzymsubstitution wahrscheinlich falsch-positiv, zumal jede Kontamination der Stuhlprobe mit Wasser oder Flüssigkeit deren Konzentrationen im Stuhl verringert [3]. Auf der anderen Seite ist wichtig zu erwähnen, dass die Prävalenz der exokrinen Pankreasinsuffizienz, welche als Folge der altersbedingten Organatrophie des Pankreas auftritt, mit einer Prävalenz von 16 % bei den über 80-jährigen Patienten angegeben wird und insbesondere bei unklaren Fällen nicht zu unterschätzen ist [4].

Eine ungewollte Gewichtsabnahme im höheren Alter ist immer ein Alarmzeichen

Eine Gewichtsabnahme in höherem Alter ist meistens ungewollt und nahezu immer irreversibel [5]. Bei der etwas selteneren, gewollten Gewichtsabnahme im höheren Alter zeigen Studien, dass ein Großteil des verlorenen Gewichts und die damit verbundenen gesundheitlichen Vorteile – insbesondere bei fortgesetzten gesunden Verhaltensweisen und körperlicher Aktivität zur Erhaltung der Muskelmasse – langfristig erhalten bleiben können [6, 7].

Die ungewollte Gewichtsabnahme bei hochaltrigen Menschen ist ein ernst zu nehmendes Alarmzeichen und fast immer als kritisch zu bewerten, da sie häufig auf eine zugrunde liegende, potenziell schwerwiegende körperliche oder psychische Ursache hinweist. Dies führt zu einem Teufelskreis aus Muskelabbau (Sarkopenie), Gebrechlichkeit (Frailty), erhöhter Infektanfälligkeit, verminderter Mobilität und einer deutlich schlechteren Prognose.

## Fazit für die Praxis

Ungewollter Gewichtsverlust bei geriatrischen Patienten ist ein ernstes Warnsignal. Obwohl eine vollständige Reversibilität nicht immer garantiert ist, insbesondere bei fortgeschrittenem Alter und komplexen Ursachen, sind frühzeitige Diagnose und eine multimodale Therapie entscheidend.

## Data Availability

Alle dieser Arbeit zugrunde liegenden Daten sind in diesem Artikel enthalten.
